# Notes and News

**Published:** 1893-10-07

**Authors:** 


### Notes and News.

Collected and Communicated.

The visit of the Duke and Duchess of York to the Royal Infirmary, Edinburgh, and the Longmore Hospital for Incurables, on Tuesday, the 3rd inst., was a brilliant success. The cordiality of their Royal Highnesses, and the genuine interest which they evidently took in the suffering inmates, won all hearts. The Duchess of York has for years identified herself with work connected with the alleviation of suffering, and His Royal Highness the Duke's reception of two seamen of the Navy, whom he found in the wards of the infirmary constituted a pleasant feature of the visit. The Duke announced that the Queen had given her permission for the Longmore Hospital to be called in future the Royal Association for Incurables. The new Nursing Home at the Infirmary, the most complete and beautiful building of the kind in the world, gave special pleasure to their Royal Highnesses.

The opening of the present Medical Session was, as usual, the occasion of some excellent addresses at the various medical schools. It is observable that the longer curriculum is favourably regarded by the teachers, whatever other aspect it may be viewed in by students. At St. Mary's Hospital Mr. Lane pointed out the extra opportunity afforded for valuable knowledge to be acquired in the study of mental diseases and fevers, branches of medicine which previously have almost necessarily received insufficient attention in the ordinary medical education, and have been made the subjects of voluntary study on leaving the school by numbers of students, notwithstanding that they could have been better pursued under direction, and with better facilities. The present system of medical education, in so far as it does not provide for the sufficient acquirement of Latin, was found fault with by another lecturer. Taking the addresses as a whole, we observe a tendency on the part of the teachers to advocate a sound and general education as a basis for a medical career, and a generally high tone of moral cultivation. Such teaching, in bearing fruit, will act beneficially on the whole profession, and still more so on the public dependent on them.

Most admirable advice was given to the students at University College, by Mr. Bilton Pollard in his address. He exhorted them to make painful manipulations as brief as possible, determining in their minds previously their course of action, and carrying it out quickly and systematically, declaring it to be positively wrong to fumble about an injured or diseased part, expecting something to turn up by doing so. He advocated the cultivation of sympathy towards poor patients especially, and indeed to all patients, both for their sakes and for the formation of their own characters also.

Mr. Timothy Holmes' address to the students of St. George's Hospital, was based on the life and works of John Hunter, who died within the walls of the hospital one hundred years ago. The lecturer had an excellent theme upon which to base his discourse, and a splendid example to offer to the students. The various resources and interests of this remarkable man, as laid bare by the lecturer, must impress minds which already are biassed by the specialistic tendency of the science of the day to regard all versatility with distrust. Mr. Holmes was privileged to be able to illustrate that true education of necessity is a wide embracing process, and to draw attention to John Hunter's life acquirements as a testimony to this.

We welcome with especial gratification Dr. P. W. Pasteur's address at the Middlesex Hospital. We recognise in it the expressed convictions of a man who has the courage of his opinions. Dr. Pasteur is evidently firmly impressed that in medicine, as in every other calling in life, the higher faculties of the mind are to be cultivated, and a heart's devotion to work is to be aimed at rather than the mere acquirement of barely sufficient knowledge to pass examinations, the standard of which only represents the minimum of that knowledge which must be possessed to ensure safety in the practice of medicine. Dr. Pasteur dwelt on the fallacy of the principle entertained by so many students that knowledge was to be taught rather than learnt, and that it was something, moreover, to be acquired with the minimum of personal exertion on the part of the student.

Sir James Crichton Browne's address to the Sheffield School of Medicine was a remarkable and masterly dissertation, using his platform as an opportunity to take his stand on the side of those amongst his fellow creatures who do not believe that all the laws of social and moral development inevitably tend to the degeneracy and decay of the Aryan race following on a period of colourless monotony. He argued against the teaching of Mr. Pearson and other modern socialists by prefacing his criticism upon them by tracing the history of the natural development of sentiency, which history goes against the theory of dead level prognosticated by such teachers as Mr. Pearson. The whole address is profoundly interesting, and should cause the younger men entering on the paths of science to pause and think, whilst his criticism on the writings of those who arrive at their conclusions "after a long course of desultory and miscellaneous reading," should warn those of the modern "advanced school" that fools step in where angels fear to tread.

